# The human trophoblast cell-surface antigen 2 (TROP-2) expression on metastasized breast cancer

**DOI:** 10.1007/s00432-026-06551-4

**Published:** 2026-07-17

**Authors:** M. Weich, L. Christl, M. Kiesel, J. Salmen, A. Wöckel, J. Diessner, E. Gerhard-Hartmann, T. Schlaiss

**Affiliations:** 1 Department of Gynaecology and Obstetrics, University of Würzburg, Josef-Schneider-Str. 4, 97080 Würzburg, Germany; 2 Institute of Pathology, University of Würzburg, Versbacher Str. 3, 97078 Würzburg, Germany; 3 Institute of Forensic Medicine, University of Würzburg, Versbacher Str. 3, 97078 Würzburg, Germany; 4 Institute of Pathology, University of Würzburg, Josef-Schneider-Str. 2, 97080 Würzburg, Germany

**Keywords:** TROP-2, H-score, Longitudinal TROP-2 expression, Breast cancer, Sacituzumab govitecan

## Abstract

**Purpose:**

The antibody–drug conjugates (ADCs) sacituzumab govitecan (SG) and datopotamab deruxtecan target trophoblast cell surface antigen 2 (TROP-2) and have shown significant efficacy in HER2-negative metastatic breast cancer (mBC). As TROP-2 may serve as a target across multiple treatment lines, detailed information on TROP-2 expression over time is of great interest.

**Methods:**

TROP-2 expression was analyzed in breast cancer (BC) samples from patients treated at the University Hospital Würzburg between 2004 and 2025 at clinically indicated biopsy time points (TP), and, in a subset, before and after SG treatment. Expression was assessed immunohistochemically using the H-score (range 0–300).

**Results:**

We evaluated 229 samples from 76 patients. Overall, patient-level TROP-2 expression was high (mean H-score 241.3 ± 73.2) and largely stable over time (TP1: 235.7 ± 60.8, n = 76; TP2: 244.6 ± 77.4, n = 72; TP3: 245.3 ± 84.4, n = 39; TP4: 244.9 ± 95.6, n = 8). However, 21 patients exhibited a decline in TROP-2 expression during disease progression, defined as a decrease of ≥ 50 H-score points between two consecutive TPs. Among 13 patients with paired biopsies obtained pre- and post-SG treatment, the mean TROP-2 H-score decreased from 227.0 ± 74.6 before SG to 202.9 ± 99.4 after SG (mean change − 24.1 ± 86.0; median 1.7, range − 170 to 110; Wilcoxon p = 0.542). TROP-2 H-score decreased in 6/13 patients and increased in 7/13 patients. Exploratory PFS analysis showed no statistically significant difference by post-SG TROP-2 change (log-rank p = 0.526).

**Conclusion:**

TROP-2 expression was generally high and relatively stable; however, some patients showed declining levels over time.

## Introduction

Over the last decade, the number of targeted treatment options for patients with breast cancer (BC) has significantly expanded, enabling increasingly individualized treatment strategies. Beyond established endocrine and anti-HER2 (human epidermal growth factor receptor-2)-directed therapies, additional potent approaches, including molecularly targeted therapies, immunotherapy, and antibody–drug conjugates (ADCs), have been developed and are in clinical practice (Leitlinienprogramm Onkologie (Deutsche Krebsgesellschaft and AWMF) 2025).

Human trophoblast cell surface antigen 2 (TROP-2) is a transmembrane glycoprotein encoded by the TACSTD2 gene and expressed in various epithelial cancers, particularly in most BC subtypes, whereas its expression is low or restricted in most normal adult epithelial tissues. TROP-2 is commonly overexpressed in triple-negative breast cancer (TNBC) and hormone receptor (HR)-positive/HER2-negative (HR + /HER2 −) BC, making it an appealing broad therapeutic target. Functionally, TROP-2 acts as a membrane signal transducer, activating intracellular pathways that can promote tumor growth and metastasis (Zhang et al. 2025).

Sacituzumab govitecan (SG) is a TROP-2-directed ADC composed of an antibody targeting TROP-2 coupled to SN-38, a topoisomerase I inhibitor. Following antigen binding and internalization, SN-38 is released intracellularly, leading to cell death. The consecutive release of the cytotoxic payload can additionally affect nearby cells and provoke their death, a phenomenon known as the bystander effect.

In the ASCENT trial (ClinicalTrials.gov identifier: NCT02574455), SG was evaluated against standard chemotherapeutic treatments of the physician’s choice (TPC), including eribulin, vinorelbine, capecitabine, or gemcitabine, in patients with metastatic TNBC (mTNBC). Median progression-free survival (PFS) was 5.6 months with SG versus 1.7 months with chemotherapy. Overall survival (OS) was significantly increased to 12.1 months with SG versus 6.7 months with standard treatment. Treatment-related adverse events included neutropenia (51%), anemia (8%), and diarrhea (10%) (Bardia et al. 2021). In the phase III TROPiCS-02 trial (ClinicalTrials.gov identifier: NCT03901339), SG was also compared with standard chemotherapy in HR + /HER2 − metastatic breast cancer (mBC). Here, SG improved OS to 14.4 months versus 11.2 months (Rugo et al. 2023). The safety profile of SG was comparable to that described in the ASCENT trial. Based on these data, SG has become a standard second-line treatment for patients with advanced or metastatic HER2-negative BC.

To date, several additional TROP-2-directed ADCs are in clinical development. Datopotamab deruxtecan (Dato-DXd) has a cleavable linker, deruxtecan as the payload, and a drug-to-antibody ratio of 4:1, compared with 7.6:1 in SG. Dato-DXd is currently being investigated in patients with TNBC and other solid tumors. In the randomized phase III TROPION-Breast01 trial (ClinicalTrials.gov identifier: NCT05104866) in previously treated HR + /HER2 − mBC, Dato-DXd significantly improved PFS compared with investigator’s-choice chemotherapy (Bardia et al. 2024a, 2025). These data led to the approval of Dato-DXd in HR + /HER2 − mBC. The TROPION-Breast02 trial (ClinicalTrials.gov identifier: NCT05374512) investigated Dato-DXd as a first-line treatment compared with chemotherapy in patients with mTNBC who are not eligible for immunotherapy and demonstrated significant improvements in PFS and OS with Dato-DXd (Dent R.A., 2025).

The comparative efficacy of SG and Dato-DXd remains to be determined, and combination strategies of TROP-2 ADCs with other targeted treatments are under investigation. In mTNBC, combining SG and the checkpoint inhibitor pembrolizumab has shown a clinically meaningful improvement in PFS in the ASCENT-04/KEYNOTE-D19 trial (ClinicalTrials.gov identifier: NCT05382286) (Tolaney et al. 2026). Further studies, including the SASCIA trial (ClinicalTrials.gov identifier: NCT04595565), are currently evaluating the use of SG in combination with immune checkpoint inhibition at earlier stages of the disease (Marme, 2021).

Receptor conversion, particularly of HER2 or HR, is a well-recognized phenomenon in mBC and may compromise the efficacy of targeted therapies (Stueber et al. 2018). Thus, reevaluation of tumor targets is good clinical practice in case of poor response based on staging examinations. In contrast, information on the possible clinical relevance of potential dynamic TROP-2 expression is limited. Preclinical data indicate that silencing or knocking out TROP-2 impairs TNBC cell proliferation (Aslan et al. 2021). However, TROP-2 assessment was not mandatory in the ASCENT trial (ClinicalTrials.gov identifier: NCT02574455), and to date, no recommendations for determining TROP-2 expression prior to or concurrent with SG treatment exist. Although differences in TROP-2 expression levels and corresponding response trends were noted, treatment is currently recommended irrespective of TROP-2 expression level (Bardia et al. 2024b).

Given the increasing use and options of TROP-2-directed ADCs for patients with BC, a better understanding of TROP-2 expression levels in the course of the disease, especially before and after therapy, may be clinically relevant for sequencing strategies and rational ADC selection. Because prospectively scheduled serial biopsies at uniform treatment-line intervals are rarely available in routine mBC care, this study was designed to capture clinically indicated biopsy episodes obtained for diagnosis of metastatic disease, confirmation of progression, receptor reassessment, or before/after relevant treatment changes. We therefore evaluated TROP-2 expression in 229 tumor samples from 76 patients with BC at different time points, including 13 patients with samples available both prior to and after SG treatment. Accordingly, the present analysis was intended to describe real-world longitudinal TROP-2 expression patterns rather than to define treatment-line-specific effects.

## Methods

### Study design and inclusion criteria

We retrospectively analyzed samples from patients with advanced or metastasized breast cancer diagnoses treated at the University Hospital of Würzburg between 2004 and 2025. The study protocol was reviewed by the Ethics Committee of the University Hospital Würzburg, which approved the retrospective analysis (Reference No. 2023012301).

Inclusion criteria were Stage IV breast cancer, histological confirmation of the primary tumor, and distant metastases. Patients with at least one evaluable sample were included in the descriptive baseline cohort; patient-level longitudinal analyses were restricted to patients with at least two distinct evaluable time points. In this retrospective design, biopsy episodes were considered clinically relevant when obtained for initial diagnosis or confirmation of metastatic disease, evaluation of progression, receptor reassessment, or before/after a systemic treatment change. Clinical data were reviewed and completed from patients’ medical records, including the location of metastasis, biopsy method, surgical resection, pathology reports, previous lines of systemic therapy, and, where applicable, SG exposure. Data were extracted from the hospital’s electronic data system (SAP), the Picture Archiving and Communication System (PACS), and “ViewPoint”. Analysis of the overall population included menopausal status, age at diagnosis, histological grading of the tumor, BC subtypes, surgical interventions, type of adjuvant and palliative treatment, localization of distant metastasis, time to relapse, and date of last contact or death. Follow-up data for these patients were collected from the national cancer register of the Lower Franconian region Würzburg (Germany), including the time of recurrence and overall survival.

Biopsy time points (TP) were assigned chronologically at the patient level. Importantly, TP labels were assigned according to chronological biopsy episodes rather than a uniform number of previous therapy lines. TP1 was defined as the first evaluable biopsy available within the metastatic-disease dataset and could therefore represent either primary/local tumor tissue or metastatic tumor tissue. TP2, TP3, and TP4 were defined as subsequent clinically indicated biopsies obtained during the course of the disease. They were not linked to a uniform calendar interval or to a predefined treatment line, although many later biopsies were obtained at progression, reassessment, or before/after a relevant treatment change. If multiple biopsies were obtained within the same diagnostic episode or clinical treatment window, they were assigned to the same TP. Consequently, the number of biopsies could exceed the number of patient-level time points. Taken together, all investigated time points were linked to treatment or disease history, but their exact timing and therapy-line context differed between patients. Patients with only one available time point were retained in the descriptive cohort but excluded from analyses requiring paired longitudinal time points.

### Histological and immunohistological methods

Histological sections were cut and stained with hematoxylin & eosin for routine histological evaluation. All immunohistochemical (IHC) stainings were performed on formalin-fixed, paraffin-embedded (FFPE) tissue slides according to the manufacturers’ instructions and standard protocols using automated immunostainers.

#### Hormone receptor status/HER2-expression

Hormone receptor (HR) status was determined by estrogen receptor (ER) and progesterone receptor (PR) immunohistochemistry (clone SP1 and 1E2, respectively, both Roche Diagnostics, Mannheim, Germany), which was performed on an automated immunostainer (Benchmark Ultra; Roche/Ventana, Tucson AZ, USA) and evaluated using the immunoreactive score (IRS) described by Remmele & Stegner (Remmele and Stegner 1987). HR positivity was defined as ER (IRS) > 0 and/or PR (IRS) > 0, and HR negativity required both ER (IRS) = 0 and PR (IRS) = 0. HR status was considered missing/unknown if ER/PR could not be determined in a structured form.

HER2 status was assessed according to established American Society of Clinical Oncology/College of American Pathologists (ASCO/CAP) guidelines by immunohistochemistry and, when indicated, fluorescence in situ hybridization (FISH) (Wolff et al. 2018, 2023). In detail, IHC staining for HER2 was performed with the clone 4B5 (Roche Diagnostics) according to the appropriate protocols on an automated immunostainer (Benchmark Ultra; Roche/Ventana). HER2 fluorescence in situ hybridization was performed using a HER2-specific probe and a centromeric probe (Kreatech™ FISH probes ERBB2 (17q12)/SE 17, Leica Biosystems, Germany) according to standard protocols and the manufacturer’s instructions.

#### TROP-2 expression

TROP-2 immunohistochemistry (clone B9, 1:500, Santa Cruz, Dallas, Texas, USA) was performed according to the manufacturers’ instructions and standard protocols using an automated immunostainer (BOND-III, Leica Biosystems, Nussloch, Germany). Semi-quantitative evaluation of IHC staining was performed using a multiplication score, specifically the H-score, by E.G.-H. and L.C. (McCarty et al. 1986). In brief, staining intensity and the percentage of positive cells were evaluated and summed according to the following formula: 1 × (% weakly positive cells) + 2 × (% moderately positive cells) + 3 × (% strongly positive cells), resulting in a range of 0 to 300. H-scores > 200 were arbitrarily defined as high expression, 101–200 as moderate, 50–100 as low, and < 50 as very low, broadly corresponding to commonly used categorical H-score approaches in TROP-2 breast cancer studies (Bardia et al. 2024b). However, no validated threshold for a clinically meaningful longitudinal change in TROP-2 H-score has been established. Therefore, a decrease of ≥ 50 H-score points was chosen as an exploratory descriptive threshold for a marked decline, because it exceeds minor scoring fluctuations and represents approximately one-sixth of the total 0–300 H-score range. This threshold was not used to define eligibility for SG treatment and did not replace the paired continuous analyses.

As mentioned above, biopsy time points were assigned chronologically at the patient level. For TROP-2 analyses, if more than one biopsy was available for a given TP, the representative patient-level value was defined as the arithmetic mean H-score across all biopsies from the individual patient assigned to that time point. Accordingly, in the patient-level longitudinal analysis, each patient contributed one representative H-score per TP, calculated as the mean H-score whenever multiple biopsies were available at that time point. For the SG paired analysis, ‘pre-SG’ was defined as any evaluable biopsy obtained prior to the first administration of SG, whereas ‘post-SG’ referred to any evaluable biopsy obtained after SG exposure. If multiple biopsies were available within either the pre-SG or post-SG window, the representative patient-level value was defined as the mean H-score in the respective window, analogous to the longitudinal TP analyses. In addition, the anatomical origin of the representative last pre-SG and first post-SG biopsy was reviewed descriptively.

## Statistical analysis

All statistical analyses were performed retrospectively using routinely collected clinicopathological data. Analyses were conducted in Python using standard statistical libraries (SciPy and statsmodels). Continuous variables are summarized as mean ± standard deviation (SD) and/or median (range), and categorical variables as counts and percentages. All tests were two-sided, and p-values < 0.05 were considered statistically significant.

To evaluate whether TROP-2 H-scores differed across serial biopsies, we performed a within-patient repeated-measures analysis using the Friedman test (nonparametric test for repeated measures). This analysis was restricted to patients with evaluable H-scores at all included time points (TP1–TP3; n = 39). For patients with more than one biopsy within the same time point, the mean H-score per patient/time point was used. Where post hoc testing was required, pairwise Wilcoxon signed-rank tests were performed for TP1 vs TP2, TP1 vs TP3, and TP2 vs TP3, with Bonferroni adjustment for multiple comparisons. TP4 was available for only a small subset of patients and was therefore summarized descriptively.

For patients with paired biopsies obtained pre- and post-SG (n = 13), within-patient changes in TROP-2 H-score were assessed using the two-sided Wilcoxon signed-rank test. If multiple biopsies were available within the pre-SG or post-SG window, the primary analysis used the arithmetic mean H-score per patient/window. As a descriptive sensitivity analysis, the last available pre-SG biopsy and the first available post-SG biopsy were additionally reviewed, with the anatomical origin of each specimen noted. A paired t-test was additionally performed as a sensitivity analysis. Normality of paired differences was assessed using the Shapiro–Wilk test. The ≥ 50-point threshold was used only to describe marked longitudinal decreases, not as the primary statistical cutoff for paired testing.

To address the possibility of spatial sampling effects, TROP-2 H-scores were compared between local/primary tumor samples and metastatic samples at the sample level using the Mann–Whitney U test. In addition, a paired patient-level comparison was performed in patients with both local/primary and metastatic tissue, using the mean H-score for each tissue category and each patient. For receptor-conversion analyses and marked TROP-2 declines, the anatomical origin of the paired biopsy episodes was summarized descriptively as local/primary versus metastatic and by organ category.

To assess changes in receptor status from baseline to follow-up, two complementary definitions were used: an any-follow-up analysis, in which follow-up was defined as any subsequent biopsy (TP > 1), and a last-follow-up analysis, in which follow-up was defined as the last available biopsy time point (TP2–TP4). HR status was defined as HR-positive if ER and/or PR IRS > 0, and HR-negative if ER and PR were evaluable and both IRS = 0. Patients with missing or unevaluable HR status at baseline and/or follow-up were excluded. HR status was evaluable in 67/72 patients in the any-follow-up analysis and in 65/72 patients in the last-follow-up analysis. Pairwise HR transition analyses were evaluable in 63 patients for TP1 → TP2, 32 patients for TP2 → TP3, and 8 patients for TP3 → TP4. HR transitions were categorized as HR loss (HR +  → HR −), HR gain (HR −  → HR +), stable HR positivity (HR +  → HR +), and stable HR negativity (HR −  → HR −). Paired changes in binary HR status were tested using the two-sided exact McNemar test. These receptor-status analyses were performed as contextual analyses of cohort heterogeneity and potential sampling bias, not as primary endpoints of the TROP-2-focused study.

PFS on SG was analyzed using the Kaplan–Meier method. SG-PFS was defined as the time from SG initiation to the first documented progression or death from any cause; patients without an event were censored at the date of last follow-up. In the subgroup with paired biopsies obtained pre- and post-SG (n = 13), patients were stratified according to the longitudinal change in TROP-2 expression in the primary mean-window SG analysis as drop (post-SG mean H-score < pre-SG mean H-score) versus steady/increase (post-SG mean H-score ≥ pre-SG mean H-score). This PFS stratification used any decrease rather than the exploratory ≥ 50-point threshold, because the SG subgroup was small and because no validated longitudinal TROP-2 H-score cut-off exists. For this stratification, pre- and post-SG values were based on the last available pre-SG biopsy and the first available post-SG biopsy, whereas the primary paired SG analysis used the mean H-score per patient/window. Survival curves were compared using the two-sided log-rank test, and observed median PFS durations were reported descriptively for each group. No multivariable analysis was performed in this subgroup because of the limited sample size.

## Results

### Patient characteristics/clinicopathological data

We analyzed 229 tumor samples from 76 patients with mBC, collected at clinically indicated time points (TP1–TP4, depending on availability). The individual TPs represented clinically relevant diagnostic or reassessment events throughout the disease course. This included diagnosis or confirmation of metastatic disease, disease progression, receptor status reassessment, and biopsies performed before or after a change in systemic treatment. Accordingly, the TPs were linked to treatment and disease history, but did not correspond to uniform treatment-line numbers across all patients. In 72/76 (94.7%) patients, samples were available from at least two distinct time points (TP1 and ≥ TP2), and these patients formed the longitudinal patient-level cohort. Four patients had only a single evaluable TP1 sample and were retained for baseline and overall descriptive analyses but excluded from analyses requiring paired longitudinal time points. In 39/76 (51.3%) patients, a third time point (TP3) was available; 8/76 (10.5%) patients had a TP4 biopsy. Overall, 17/76 (22.4%) patients had ≥ 4 biopsies over the course of their disease; however, several of these biopsies were from the same clinical episode/time point and were averaged for patient-level analysis. The difference between the number of patients with ≥ 4 biopsies and the number with TP4 is explained by repeated biopsies assigned to the same patient-level time point. The maximum interval between the first and last biopsy was 17 years.

At TP1, local/primary tumor tissue was investigated in 41/76 (53.9%) of patients and metastatic tumor tissue in 35/76 (46.1%). In the SG-paired subgroup, TP1 was represented by local/primary tumor tissue in 5/13 (38.5%) and by metastatic tissue in 8/13 (61.5%) patients. Receptor subtype at TP1 was HR + /HER2 − (45/76, 59.2%), TNBC (16/76, 21.1%), HR + /HER2 + (9/76, 11.8%), HR − /HER2 + (2/76, 2.6%), and HR/HER2 unknown (4/76, 5.3%). One patient-level HER2-borderline case at TP1 (1/76, 1.3%) was classified as HER2 − and included in the HR + /HER2 − subgroup. Among patients with local/primary tumor tissue at TP1, 40/41 (97.6%) had metastatic tissue sampling during follow-up. Metastatic sites were most commonly recorded in bone (46/76, 60.5%), lymph nodes (40/76, 52.6%), liver (39/76, 51.3%), and lung/pleura (34/76, 44.7%), followed by skin (13/76, 17.1%), CNS (4/76, 5.3%), and chest wall (3/76, 3.9%) (Table 1).

**Table 1:** Clinicopathological characteristics of the overall cohort and of the subgroup with paired TROP-2 IHC at TP1 and pre-/post-SG treatment with sacituzumab govitecan (SG). HER2 borderline cases were classified as HER2 − ; at TP1, one patient-level HER2-borderline case was included in HR + /HER2 −

Clinicopathological characteristic	Overall cohort (TP1 baseline)	Paired TP1 subgroup (≥ 2 TP1 samples)	SG paired subgroup
Patients, n	76	23	13
Samples, n	229	83	50
Age at diagnosis (TP1), years; median (range)	52.0 (27.1–71.8)	55.5 (37.9–67.1)	43.1 (32.4–71.4)
TP1 biopsy origin: local/primary, n (%)	41 (53.9%)	11 (47.8%)	5 (38.5%)
TP1 biopsy origin: metastatic, n (%)	35 (46.1%)	12 (52.2%)	8 (61.5%)
Specimens before/after SG, n	–	–	37 / 13
Representative last pre-SG biopsy obtained during metastatic disease course, n (%)	–	–	13 (100%)
Representative first post-SG biopsy obtained during metastatic disease course, n (%)	–	–	13 (100%)
HR/HER2 status at time point 1			
HR + /HER2 −	45 (59.2%)	15 (65.2%)	7 (53.8%)
TNBC	16 (21.1%)	4 (17.4%)	4 (30.8%)
HR + /HER2 +	9 (11.8%)	4 (17.4%)	0 (0.0%)
HR − /HER2 +	2 (2.6%)	0 (0.0%)	1 (7.7%)
HR + /HER2 − subset: HER2 borderline classified as HER2 −	1 (1.3%)	0 (0.0%)	0 (0.0%)
HR/HER2 unknown	4 (5.3%)	0 (0.0%)	1 (7.7%)
TROP-2 H-score at time point 1 – median (range)	245.0 (24–300)	257.5 (115–300)	210.0 (24–300)
TROP-2 H-score at time point 1 – mean ± SD	235.7 ± 60.8	254.9 ± 44.6	210.3 ± 82.4
TROP-2 H-score pre-SG (paired cases), mean ± SD	–	–	227.0 ± 74.6
TROP-2 H-score post-SG (paired cases), mean ± SD	–	–	202.9 ± 99.4
Δ H-score (after–before), mean ± SD	–	–	− 24.1 ± 86.0
Δ H-score (after–before), median (range)	–	–	1.7 (− 170–110)
Primary metastatic disease at baseline (time point 1), n (%)	36 (47.4%)	13 (56.5%)	8 (61.5%)
Metastatic sites (patient-level presence)			
Liver	39 (51.3%)	10 (43.5%)	10 (76.9%)
Bone	46 (60.5%)	14 (60.9%)	9 (69.2%)
Lung/pleura	34 (44.7%)	10 (43.5%)	9 (69.2%)
CNS	4 (5.3%)	3 (13.0%)	0 (0.0%)
Lymph node	40 (52.6%)	9 (39.1%)	10 (76.9%)
Skin	13 (17.1%)	5 (21.7%)	3 (23.1%)
Chest wall	3 (3.9%)	2 (8.7%)	1 (7.7%)

### Contextual HR/HER2 receptor-status analyses

As the primary objective of this study was the longitudinal assessment of TROP-2 expression, changes in receptor-status are presented here solely to provide contextual information and to address potential sampling effects. Detailed transition metrics are provided in Supplementary Tables S1 and S2.

Analysis of potential receptor status changes from baseline (TP1) to follow-up was performed in patients with ≥ 2 time points (n = 72) using two complementary definitions. In the any-follow-up analysis (TP1 vs any subsequent biopsy), HR status was evaluable in 67/72 patients, HR loss (HR +  → HR −) occurred in 4/67 (6.0%), and HR gain (HR −  → HR +) in 7/67 (10.4%). In the last follow-up analysis (TP1 vs the last available biopsy), HR status was evaluable in 65/72 patients, HR loss occurred in 6/65 (9.2%), and HR gain in 4/65 (6.2%). Stable HR positivity and stable HR negativity in the last follow-up analysis were observed in 45/65 (69.2%) and 10/65 (15.4%) patients, respectively.

**Fig. 1:**
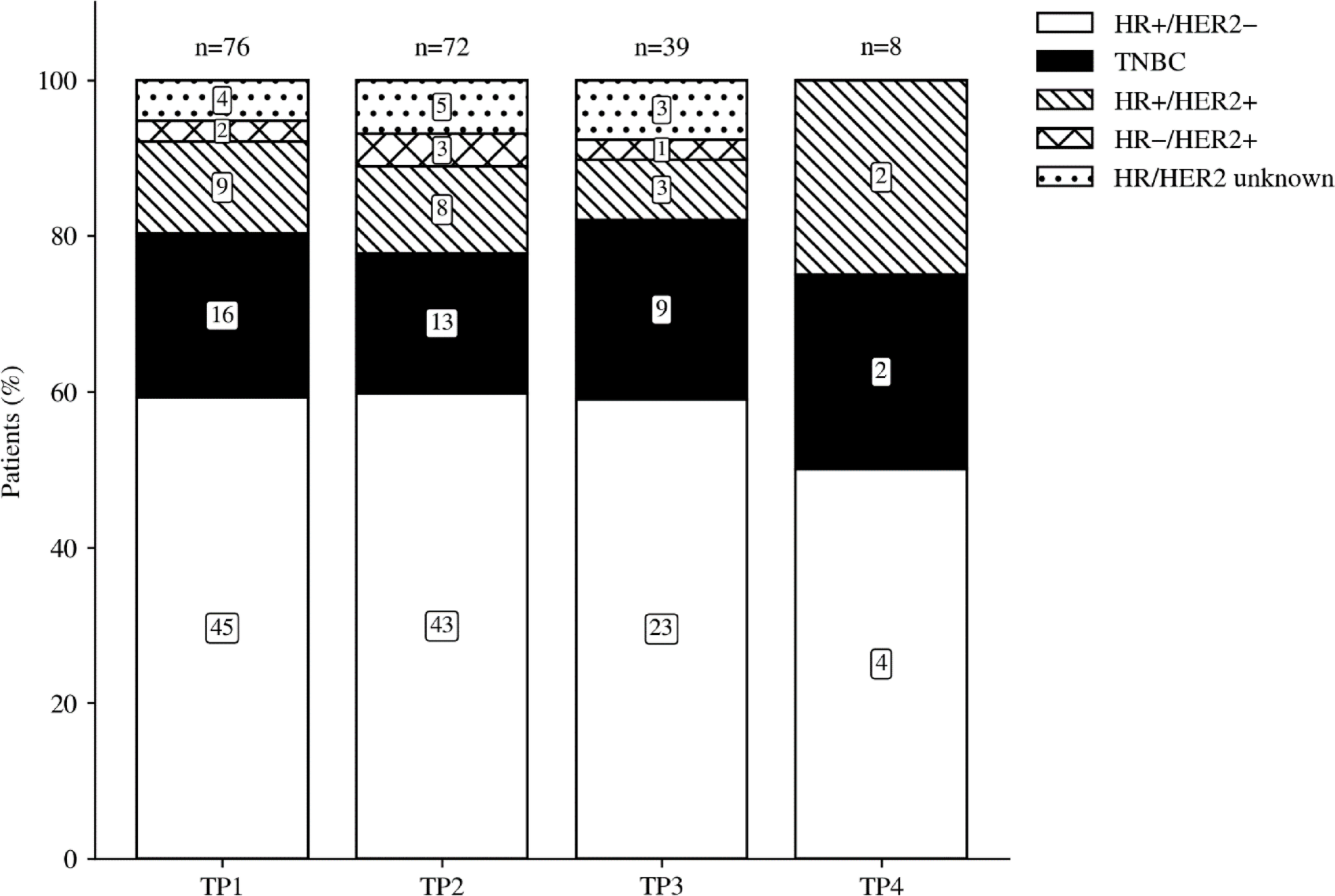

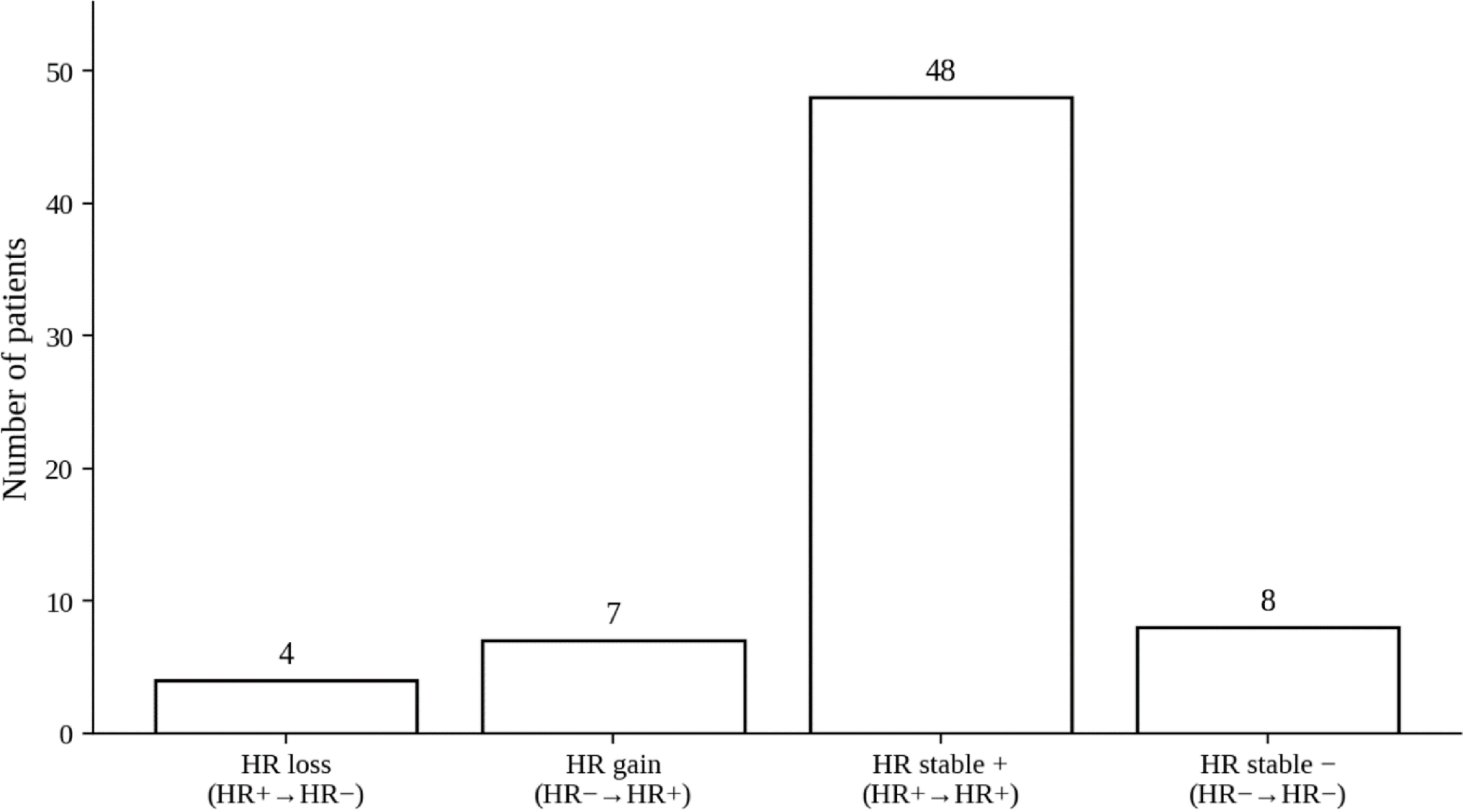

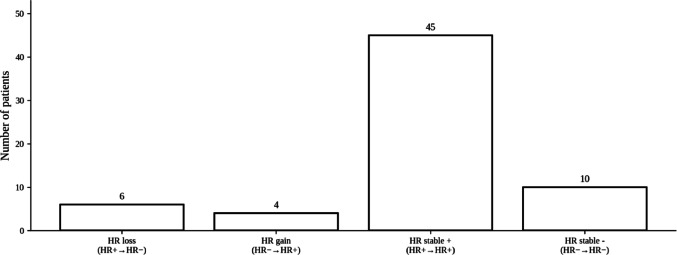
**A** HR/HER2 subtype distribution across longitudinal biopsies (TP1–TP4). For patients with multiple biopsies at the same time point, the biopsy with the most complete receptor information (ER, PR, and HER2) was selected for subtype assignment (tie-break: latest biopsy date). Stacked bars depict the percentage of patients in each HR/HER2 subtype at each time point, with absolute patient counts annotated within the bars; the total number of patients with available subtype information is shown above each bar (n). Subtypes were defined as HR + /HER2 − , TNBC, HR + /HER2 + , HR − /HER2 + , and HR/HER2 unknown; HER2 borderline cases were classified as HER2 − . **B** Change in hormone receptor (HR; ER and/or PR) status from baseline to follow-up. Baseline was defined as time point 1 (TP1) and follow-up as any subsequent biopsy (TP > 1). Bars show the number of patients with HR loss (HR +  → HR −), HR gain (HR −  → HR +), stable HR positivity (HR +  → HR +), or stable HR negativity (HR −  → HR −). Analysis included patients with ≥ 2 time points (n = 72); HR status was evaluable in 67 patients, while 5 patients had unknown HR status at TP1 and/or follow-up. Baseline HR status at TP1 was determined using a time point-level representative approach. If multiple biopsies were available at TP1 for the same patient, TP1 HR status was assigned as HR-positive if ER and/or PR were positive in any of the TP1 biopsies. TP1 was assigned as HR-negative only if all evaluable TP1 biopsies were HR-negative. TP1 was classified as unknown only when HR status was missing across all TP1 biopsies. Follow-up HR status (TP > 1) was assigned analogously using all subsequent biopsies. **C** Change in hormone receptor (HR; ER and/or PR) status from baseline to the last available follow-up biopsy. Baseline was defined as time point 1 (TP1) and follow-up as the last available biopsy at TP2–TP4. Bars show the number of patients with HR loss (HR +  → HR −), HR gain (HR −  → HR +), stable HR positivity (HR +  → HR +), or stable HR negativity (HR −  → HR −). Analysis included patients with ≥ 2 time points (n = 72); HR status was evaluable in 65 patients, while 7 patients had unknown HR status at TP1 and/or at the last follow-up. Baseline HR status at TP1 was determined using a time point-level composite approach: TP1 was assigned HR-positive if ER and/or PR were positive in any TP1 biopsy; TP1 was assigned HR-negative only if all evaluable TP1 biopsies were HR-negative; and TP1 was classified as unknown only when HR status was missing across all TP1 biopsies. Follow-up HR status at the last available time point (TP2–TP4) was assigned analogously, using all biopsies within that last time point

For HER2 (with borderline categorized as HER2 −), the any-follow-up analysis was evaluable in 67/72 patients; HER2 loss (HER2 +  → HER2 −) occurred in 5/67 (7.5%) and HER2 gain (HER2 −  → HER2 +) in 6/67 (9.0%). In the last follow-up analysis, HER2 status was evaluable in 65/72 patients, HER2 loss occurred in 5/65 (7.7%), and HER2 gain in 4/65 (6.2%), while stable HER2 positivity and stable HER2 negativity were observed in 7/65 (10.8%) and 49/65 (75.4%) patients, respectively (Fig. 1A, Supplementary Tables S1 and S2).

To explore potential sampling bias, we reviewed the anatomical source of all HR- or HER2-conversion cases in the last follow-up analysis. Among 19 total HR/HER2 conversion events, 9 occurred between local/primary and metastatic samples, and 10 occurred between different metastatic reassessment episodes; 14/19 involved a change in organ category. For HR conversion events specifically, 5/10 occurred between local/primary and metastatic samples, and 7/10 involved a different organ category. For HER2 conversion events, 4/9 occurred between local/primary and metastatic samples, and 7/9 involved a different organ category. These observations support a cautious interpretation of HR/HER2 switches, which may represent (in part) potential spatial sampling effects.

### Longitudinal TROP-2 expression (primary analysis)

**Fig. 2:**
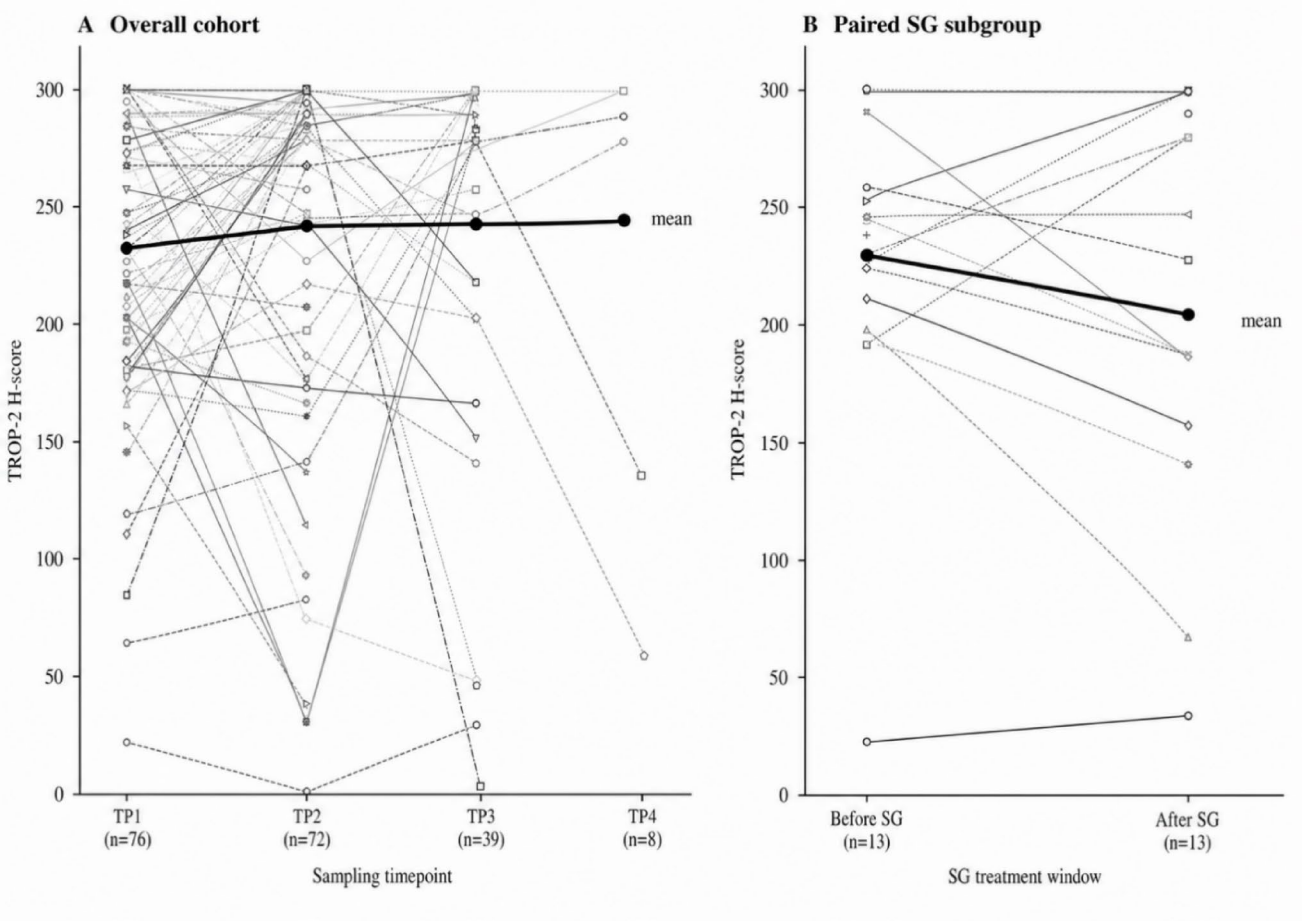

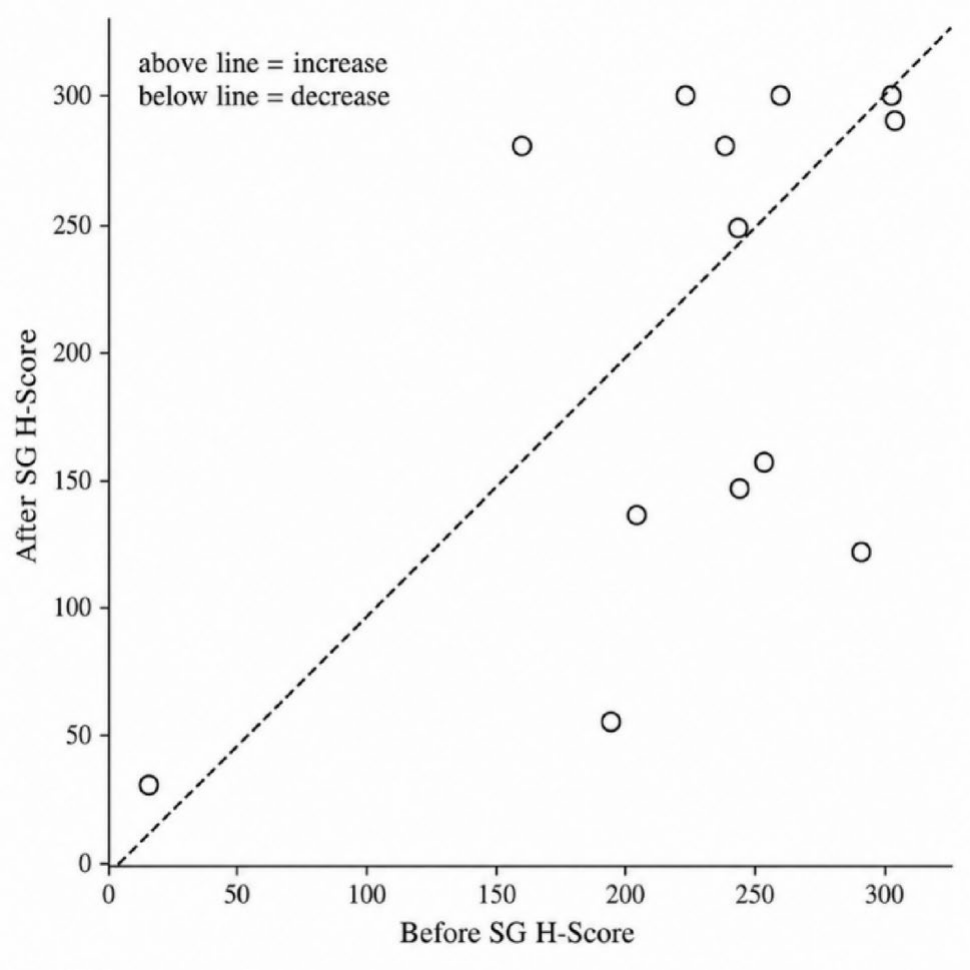

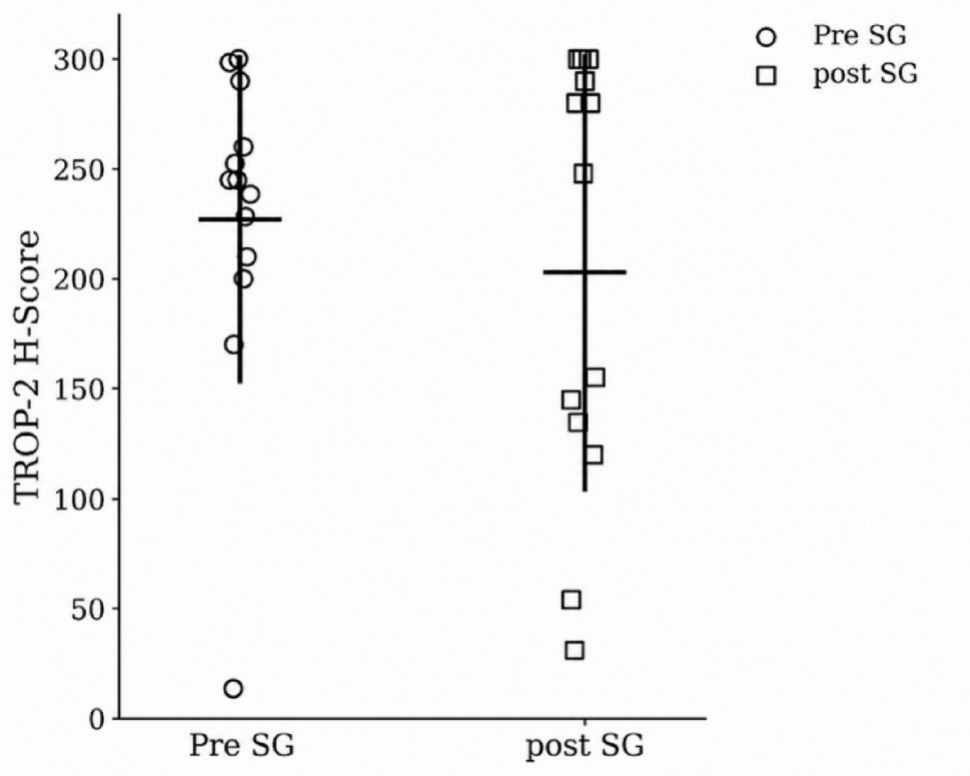
Patient-level evolution of TROP-2 H-score. **A** Patient-level evolution of TROP-2 H-score across longitudinal biopsy time points (TP) in the overall cohort (TP1–TP4). Each connected line represents one patient; when multiple biopsies were available within a given time point, the mean H-score per patient/time point was used. Black lines indicate patients with a marked decline, defined descriptively as a decrease of ≥ 50 H-score points between two consecutive time points. Filled black circles with error bars indicate mean ± SD. **B** Patient-level TROP-2 H-score before and after sacituzumab govitecan (SG) exposure in the SG-paired subgroup (*n* = 13), using the mean H-score per patient/window. Each connected line represents one patient. Black lines indicate a decrease post-SG, whereas grey lines indicate stable or increased H-score. Filled black diamonds with error bars indicate mean ± SD. Horizontal dotted reference lines denote H-score category boundaries at 50, 100, and 200. **C** Each dot represents one patient using the mean H-score per patient/window. The dashed line indicates the line of identity (y = x); points above the line indicate increased TROP-2 expression post-SG, and points below indicate decreased expression. **D** TROP-2 H-score pre- and post-SG in paired biopsies (n = 13), using the mean H-score per patient/window. Each symbol represents one patient (open circles: pre-SG; open squares: post-SG). Horizontal bars indicate the mean, and vertical lines indicate ± SD

**Fig. 3:**
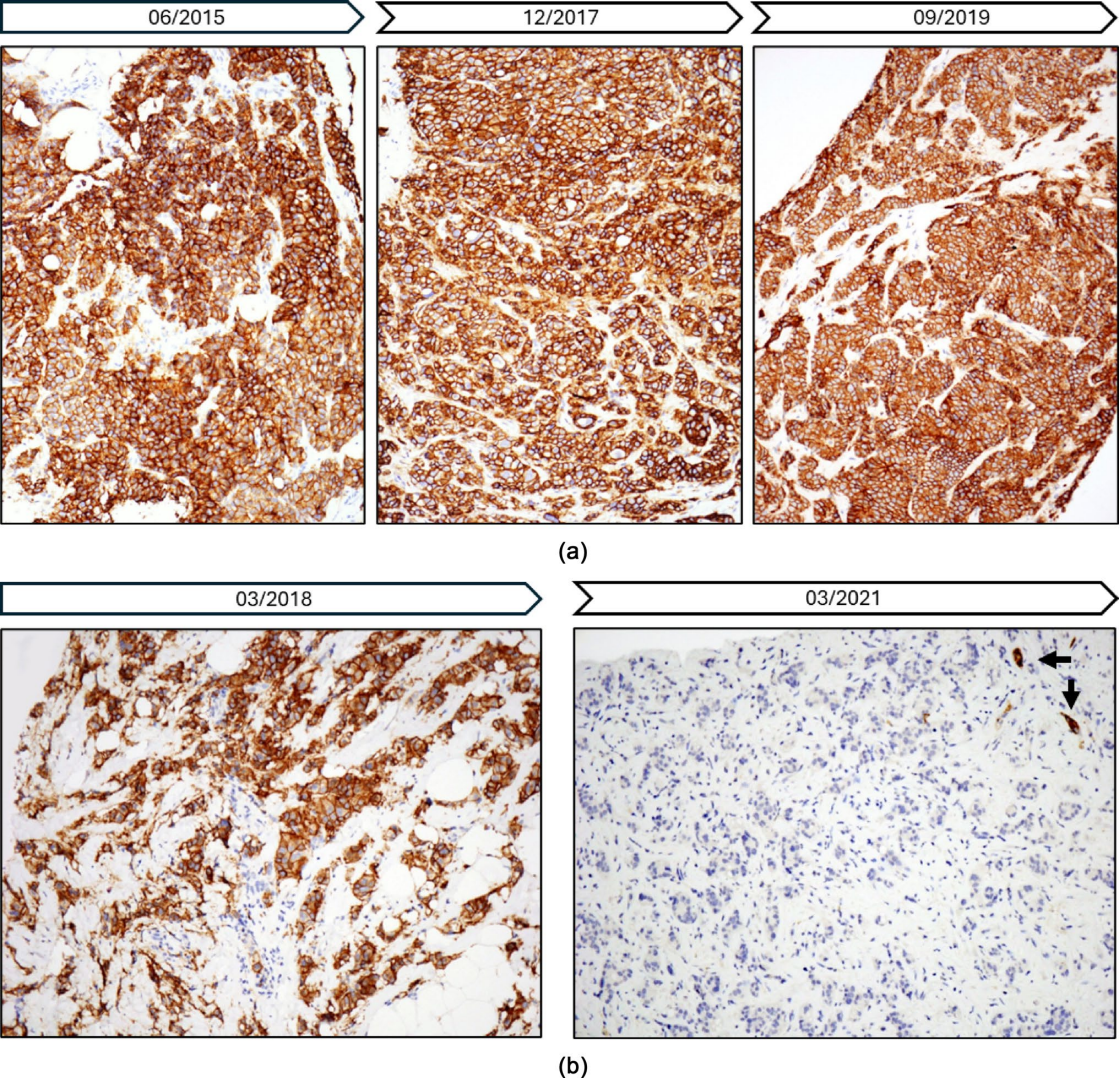

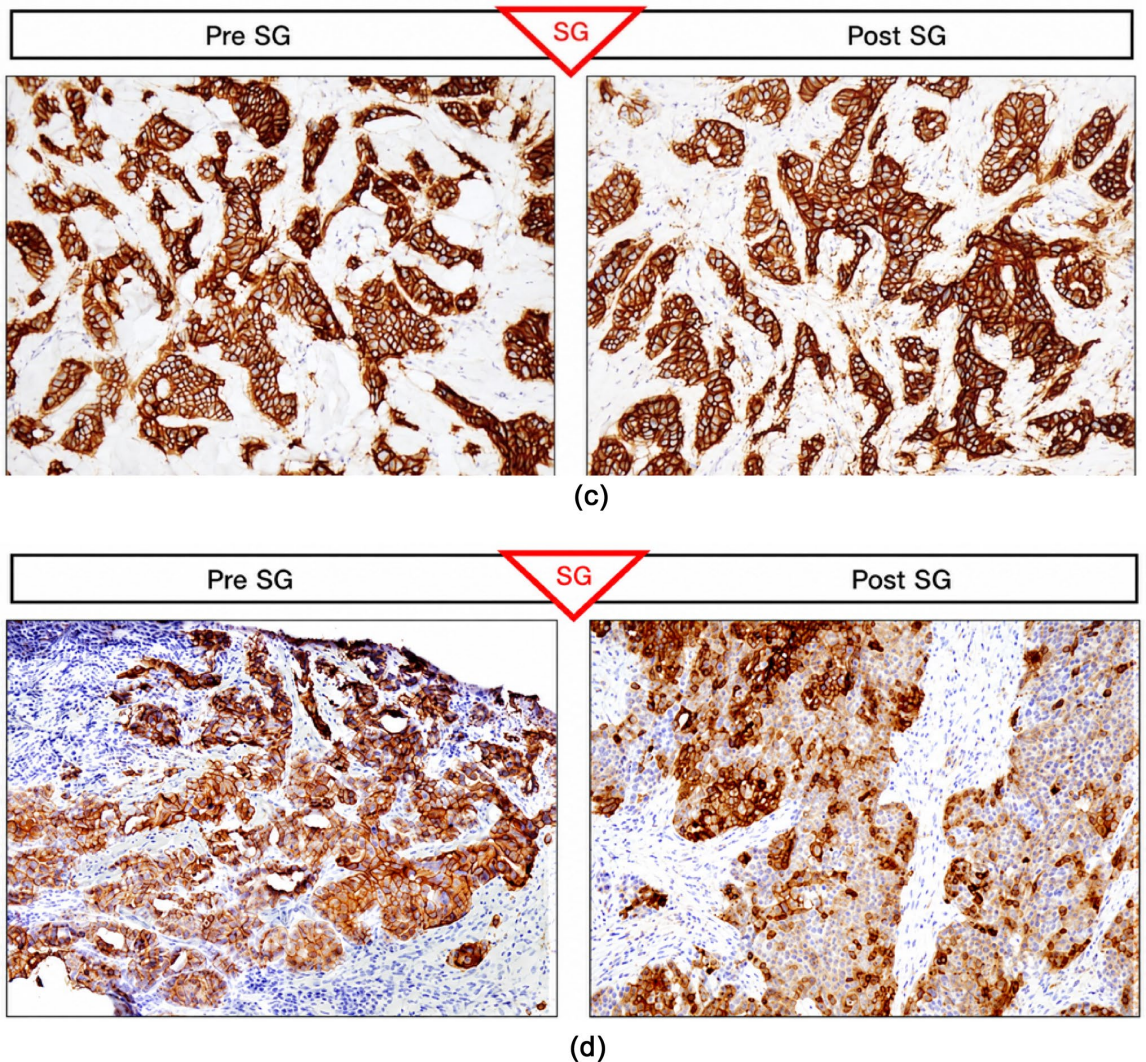
Exemplary presentation of TROP-2 protein expression evaluated by immunohistochemistry in four patients with metastatic breast cancer (mBC). **A** Stable TROP-2 expression over time. The first staining was performed on mammary tissue from 2015, at the time of diagnosis of primarily metastasized HR + BC (bone metastasis). TROP-2 expression was high (H-score 270). After receiving endocrine therapy, the patient progressed in 2017. New liver metastases were biopsied to determine the current receptor status. H-score was 300. Treatment was changed to monochemotherapy, then to cyclin-dependent kinase 4/6 (CDK4/6) inhibitor, due to HR + /HER2 − mBC. In 2019, the patient experienced further progression in the liver and underwent another liver biopsy. The H-score was still 300. The patient was treated with best supportive care and died in 2019. **B** Immunohistochemistry shows a loss of TROP-2 expression in a breast cancer patient over time. In 2018, this patient was diagnosed with metastatic HR + /HER2 − breast cancer that had spread to the bone and liver. A biopsy was taken from the liver and showed a high TROP-2 expression (H-score 300). The liver metastasis progressed under chemotherapy. In 2021, she received another liver biopsy confirming an HR + /HER2 − BC. TROP-2 expression was lost (H-score 0). The arrows indicate TROP-2 expression in the bile duct proliferates (internal positive control). **C** Pictures display immunohistochemistry of a stable TROP-2 expression before and after the treatment of mBC with sacituzumab govitecan (SG). The patient was diagnosed with early-stage TNBC in 2017. After receiving treatment according to national guidelines, she developed lung, liver, bone, and pleural metastasis until 2020. After receiving several lines of chemotherapy, the patient underwent a punch biopsy of the thoracic wall in 2021. Pathological workup confirmed mTNBC, and the patient received checkpoint inhibitor and poly (ADP-ribose) polymerase (PARP) inhibitor therapy due to tumor progression. The H-score was 295 in 2021. The patient was treated with SG from 7/2021 up until 4/2022. She showed tumor progression in the liver, lung, and cutaneous progression and underwent another biopsy in the breast. The H-score was 300. **D** Pictures display immunohistochemistry of decreasing TROP-2 expression after treatment with sacituzumab govitecan (SG) of a patient with mBC. The patient was diagnosed with primarily metastasized TNBC in 2020. In 2020, biopsy material from the breast showed high TROP-2 expression (H-score 290). After being treated with checkpoint blockade and pegylated liposomal doxorubicin, the patient progressed. She received SG from 12/2021 until 8/2022. She showed tumor progression in the lung, paracardial region, and breast. She underwent another biopsy in the breast, showing a decreased TROP-2 expression level (H-score 120)

Overall, TROP-2 H-scores were high (mean 241.3 ± 73.2; median 280; range 1–300) and remained relatively stable across time points, with mean H-scores of 235.7 ± 60.8 (TP1; n = 76), 244.6 ± 77.4 (TP2; n = 72), 245.3 ± 84.4 (TP3; n = 39), and 244.9 ± 95.6 (TP4; n = 8) (Fig. 2A). In patients with TP1–TP3 available (n = 39), the Friedman test did not indicate a statistically significant change over time (p = 0.178). Using patient-level values per time point (mean H-score per patient/time point), at TP1, 54/76 (71.1%) patients showed high expression (H-score > 200), 19/76 (25.0%) moderate (101–200), 2/76 (2.6%) low (50–100), and 1/76 (1.3%) very low (< 50). At TP2, H-score was high in 55/72 (76.4%), moderate in 11/72 (15.3%), low in 2/72 (2.8%), and very low in 4/72 (5.6%). At TP3, 32/39 (82.1%) showed a high, 3/39 (7.7%) a moderate, 2/39 (5.1%) a low, and 2/39 (5.1%) a very low TROP-2 expression according to the H-score. At TP4, high expression was observed in 6/8 (75.0%), moderate in 1/8 (12.5%), and low in 1/8 (12.5%). IHC stainings from a patient with constant TROP-2 expression over time are exemplified in Fig. 3A. Notably, one patient showed a complete loss of TROP-2 expression at TP3 (H-score 0) after previously high expression at TP1 and TP2 (H-scores 300 and 290) (Fig. 3B). In addition, 21/76 patients showed a marked decline in TROP-2 expression over the course of disease, defined as a decrease of ≥ 50 H-score points between two consecutive time points (patient-level) (Fig. 2A).

Thus, the main longitudinal finding was preserved high TROP-2 expression in most patients, while marked declines were observed only in a subset. TROP-2 expression did not differ relevantly between local/primary and metastatic tissue. At the sample level, local/primary samples (n = 65) had a mean H-score of 243.8 and a median H-score of 270.0, while metastatic samples (n = 164) had a mean H-score of 244.4 and a median H-score of 280.0 (Mann–Whitney U test p = 0.268). In the paired patient-level comparison of 40 patients with both local/primary and metastatic samples, mean H-scores were 235.8 in local/primary samples and 248.7 in metastatic samples, with a median within-patient difference of + 5 H-score points for metastases (Wilcoxon p = 0.268). Thus, metastases did not show globally higher TROP-2 levels in this cohort.

### TROP-2 expression before and after SG exposure

We further analyzed 13 patients with paired tissue samples obtained pre- and post-SG. ‘Pre-SG’ referred to biopsies obtained prior to the first administration of SG, and ‘post-SG’ referred to biopsies collected after SG exposure. In total, 37 specimens were available pre-SG and 13 specimens post-SG. According to the clinical records, SG was administered after previous systemic treatment for metastatic disease. However, the exact number and types of previous therapy lines varied between patients; therefore, this subgroup should be regarded as a clinically selected later-line population rather than a uniform treatment-line cohort. The remaining 63 patients did not contribute evaluable paired pre- and post-SG tissue and were consequently excluded from SG-specific paired analyses. Median age at TP1 in this subgroup was 43.1 years (range, 32.4–71.4), and 10/13 patients (76.9%) underwent liver biopsy sampling during the metastatic course. For the paired comparison, the representative last pre-SG and first post-SG biopsy specimens were metastatic in all 13 patients. Using the predefined mean H-score per patient/window, TROP-2 H-score showed a non-significant mean decrease from pre- to post-SG (mean ± SD: 227.0 ± 74.6 vs. 202.9 ± 99.4; Δ =  − 24.1 ± 86.0; median Δ = 1.7, range − 170 to 110; paired t-test p = 0.332; Wilcoxon p = 0.542). After SG exposure, H-scores increased in 7/13 patients (53.8%), and decreased in 6/13 (46.2%). Categorically, pre-SG H-scores were > 200 in 10/13, 101–200 in 2/13, and < 50 in 1/13; post-SG H-scores were > 200 in 7/13, 101–200 in 4/13, 50–100 in 1/13, and < 50 in 1/13 (Fig. 2B/D). In the descriptive sensitivity analysis comparing the last pre-SG and first post-SG biopsies, H-scores decreased in 6/13 patients, increased in 3/13, and remained unchanged in 4/13. Representative IHC stainings from a patient with stable TROP-2 after SG are shown in Fig. 3C, whereas Fig. 3D illustrates a reduction in TROP-2 expression after SG treatment. Analysis of the SG subgroup was considered exploratory and descriptive in nature and was not used to derive therapy-line-specific conclusions.

### Longitudinal TROP-2 decline and exploratory PFS analysis

Overall, TROP-2 expression was high and relatively stable across time points. Nevertheless, we identified a subgroup of patients showing declining TROP-2 H-scores in later biopsies obtained after multiple lines of systemic therapy, suggesting a potential dynamic regulation of TROP-2 under prolonged treatment pressure. Across the entire cohort, 21 patients (27.6%) demonstrated a marked longitudinal TROP-2 decrease, defined descriptively as a reduction of ≥ 50 H-score points between two consecutive patient-level time points. Within the SG-paired subgroup, 5/13 patients (38.5%) showed a ≥ 50-point decrease in mean TROP-2 H-score following SG exposure. As this threshold has not been clinically validated for longitudinal TROP-2 assessment, these findings were interpreted as descriptive indicators of substantial changes in expression rather than as a distinct, clinically established biomarker category.

**Fig. 4:**
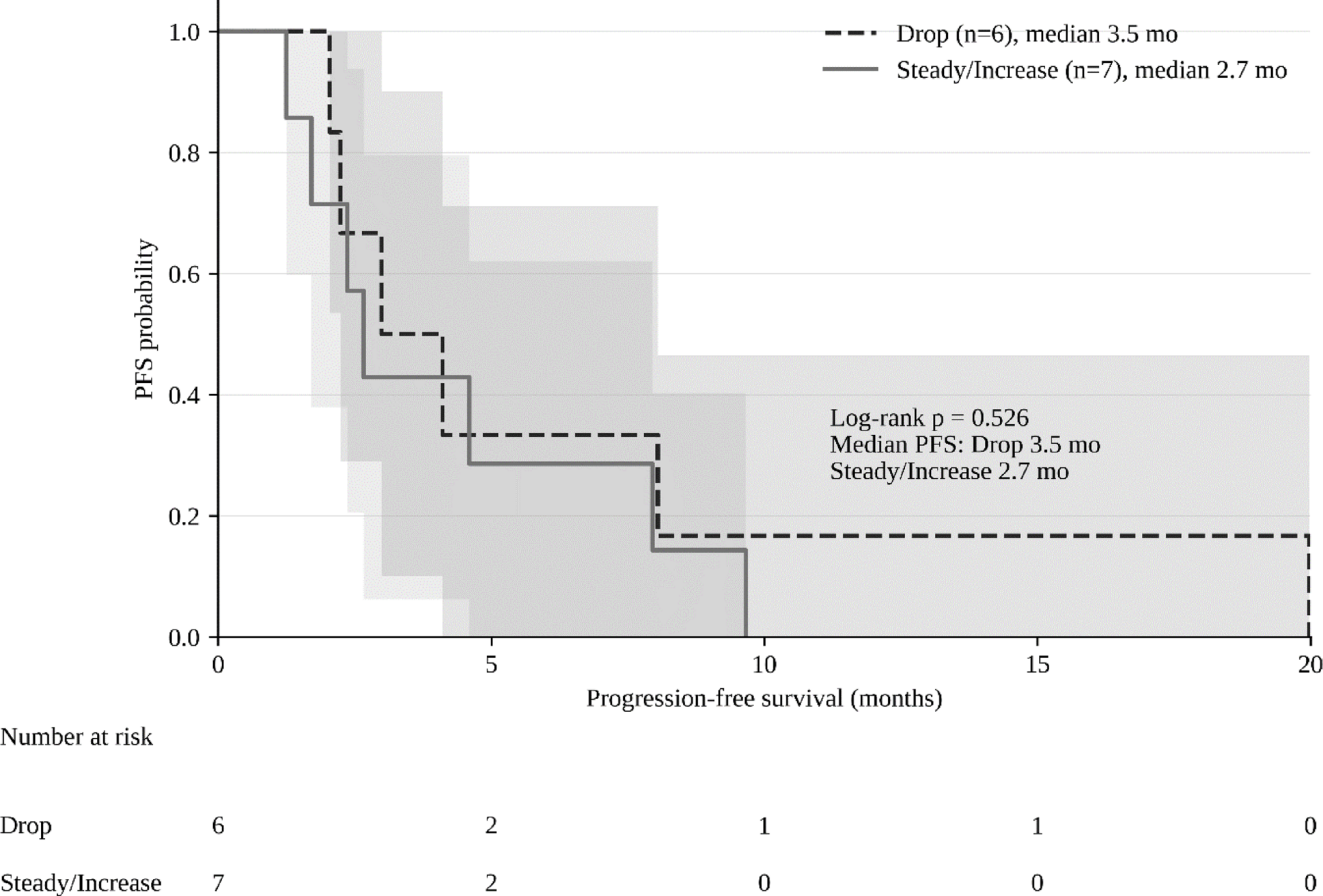
Progression-free survival on SG stratified by change in TROP-2 expression. Kaplan–Meier curves show PFS from SG start to progression or death in patients with paired biopsies obtained pre- and post-SG (n = 13). Patients were grouped as Drop (first post-SG TROP-2 H-score lower than last pre-SG; n = 6) and Steady/Increase (first post-SG H-score greater than or equal to last pre-SG; n = 7). Shaded areas indicate 95% confidence intervals. Survival curves were compared using the two-sided log-rank test (p = 0.526). Observed median PFS was 3.5 months in the Drop group and 2.7 months in the Steady/Increase group

In the exploratory PFS comparison, patients with any decrease in TROP-2 expression following SG treatment had a median observed PFS duration of 3.5 months, compared with 2.7 months in patients with stable or increased TROP-2 expression (log-rank p = 0.526; Fig. 4). This exploratory PFS comparison used the last available pre-SG and first available post-SG patient biopsy as the SG analysis and was not based on the descriptive ≥ 50-point threshold.

## Discussion

Breast cancer treatment is increasingly characterized by individualization. Besides classical therapeutic biomarkers such as HR status and HER2 expression, several additional targets have gained clinical relevance. TROP-2 is one of the targets against which potent ADCs have been developed.

Receptor discordance during the course of mBC is a well-recognized phenomenon. Numerous analyses investigating ER, PR, and HER2 have demonstrated that loss of hormone receptor expression is associated with inferior OS. The gain of ER expression was associated with longer post-recurrence survival (Shiino et al. 2022). Undetected receptor discordance may result in the administration of ineffective therapeutics, with potentially avoidable toxicity and healthcare costs. Here, we report HR and HER2 data as contextual information to illustrate biopsy heterogeneity and the potential impact of sampling bias, while the primary objective of the present study was to investigate the longitudinal expression of TROP-2.

Data regarding TROP-2 expression during the course of BC and its potential clinical relevance, as well as the optimal methodology for its assessment, remain limited. The most widely used technique is immunohistochemical analysis of TROP-2 protein expression, and the semi-quantitative H-score is commonly used for evaluation. Accordingly, in the pivotal clinical trial evaluating the efficacy of SG in HER2-negative mBC, TROP-2 expression was assessed by IHC and H-score (Bardia et al. 2021; McCarty et al. 1986). However, more elaborate methods involving computational pathology platforms have also been suggested. A quantitative continuous scoring (QCS) companion diagnostic for TROP-2 was evaluated in the Phase III TROPION-Lung01 trial (ClinicalTrials.gov identifier: NCT04656652). Tissue samples from patients with non-small cell lung cancer (NSCLC) were tested and considered TROP-2-positive if ≥ 75% of tumor cells showed a normalized membrane ratio (NMR) of ≤ 0.56, indicating a relatively higher cytoplasmic proportion of TROP-2 in the cytoplasm. While Dato-DXd reduced the risk of disease progression or death by 25% versus TPC in the overall population, the reduction was 43% in the TROP-2 QCS biomarker-positive group. These results led the authors to the assumption that analyses of TROP-2 expression at the cell membrane and inside the tumor cells need to be addressed (AstraZeneca 2024). Nevertheless, simpler approaches, such as the H-score assessment, have the important advantage that they can be quickly and cost-effectively evaluated by any pathologist without the need for additional equipment. Moreover, it seems reasonable to assume that the TROP-2 expression on the cell membrane (and not in the cytoplasm) is most relevant for the efficacy of an ADC therapy. Thus, the respective trials leading to the approval of TROP-2 ADCs in mBC used IHC and the H-score, although TROP-2 expression evaluation was not mandatory. Interestingly, in the ASCENT trial (ClinicalTrials.gov identifier: NCT02574455), which evaluated SG in patients with mTNBC, differential TROP-2 expression levels were recognized (Bardia et al. 2021). TROP-2 expression was assessable in 168 patients in the SG arm and 150 patients in the TPC arm, with the majority of tumors demonstrating medium or high expression (H-score ≥ 130). SG significantly improved median progression-free survival (PFS) and overall survival (OS) compared with TPC. Numerically better responses to SG were observed in patients with high- and medium-TROP-2-expressing tumors compared with those with low expression. However, only a marginally significant interaction between treatment effect and continuous TROP-2 expression was reported for OS. Given the limited patient numbers within subgroups and the benefit of SG across all H-score levels compared with TPC, regulatory approval and the manufacturer recommendations do not restrict treatment based on TROP-2 expression levels (Bardia et al. 2024b).

Jeon et al. evaluated TROP-2 expression in 807 TNBC patients and found no differences in H-scores in patients with or without neoadjuvant chemotherapy, nor in those with mBC. Likewise, no significant associations were observed between TROP-2 expression (high versus low) and clinicopathological characteristics. However, despite the substantial cohort size, the study did not address longitudinal changes in TROP-2 expression over time or under selective pressure from SG treatment (Jeon et al. 2022).

To date, longitudinal data on TROP-2 expression across different treatment time points and under selective therapeutic pressure remain limited. However, with the advent of several new TROP-2-targeting therapeutic options for mBC, and given that the TROP-2-directed ADC SG is a second-line or later treatment option, this is of immediate clinical interest. Thus, we here investigated the expression of TROP-2 in mBC patients at different clinically indicated time points. A major strength of our study is the longitudinal assessment of TROP-2 H-scores over treatment periods of up to 17 years, and paired analyses pre- and post-SG exposure in a subgroup of patients. The sampling time points should be understood as clinically indicated biopsy events performed for disease confirmation, progression assessment, therapeutic reassessment, or treatment modification, rather than as protocol-defined evaluations following identical therapy lines. Consistent with previous reports, we observed generally high and relatively stable TROP-2 expression levels over time, as assessed by H-score. In addition, TROP-2 levels were not globally higher in metastatic lesions compared to local/primary samples, neither in the sample-level analysis nor in the paired patient-level comparison. Notably, 21 patients showed a decrease, or even complete loss, of TROP-2 expression during the course of treatment. The exploratory threshold of a ≥ 50-point decrease in H-score was chosen to identify substantial changes beyond expected semi-quantitative scoring variability, although no validated threshold for longitudinal TROP-2 dynamics currently exists. In the subgroup with paired biopsies obtained pre- and post-SG (n = 13), TROP-2 H-scores decreased in 6/13 patients and increased in 7/13 patients in the primary mean-window analysis; notably, 5/13 showed a decrease of ≥ 50 H-score points following SG exposure. In this subgroup, ‘pre-SG’ refers to samples obtained before the first SG administration, whereas ‘post-SG’ samples were collected after SG exposure. For all 13 patients, both the representative last pre-SG and first post-SG biopsies originated from metastatic lesions, thereby reducing concerns related to primary-versus-metastatic sampling differences; however, spatial sampling effects across different biopsy sites cannot be excluded. The exploratory comparison of PFS according to post-SG TROP-2 changes was not statistically significant; therefore, this subgroup analysis should not be interpreted as evidence of a prognostic association. Potential explanations for heterogeneous H-score changes include treatment selection pressure under SG, negative selection of patients requiring later-line TROP-2-directed therapy, higher overall treatment pressure, more aggressive disease biology, receptor-subtype distribution, heterogeneous prior therapies, and spatial sampling effects across different metastatic organs. Consequently, no causal relationship between TROP-2 loss and adverse prognosis can be inferred from the present data. Nevertheless, it may be worthwhile to keep this aspect in focus in further studies. Taken together, the paired SG analysis should be regarded as exploratory and hypothesis-generating rather than as evidence that immunohistochemical H-score changes directly reflect tumor biology under SG.

TROP-2 expression can be influenced by multiple signaling pathways, including TGF-β, Wnt/β-catenin & YAP, various transcription factors, and ERK/MAPK signaling. However, “the exact role of TROP-2 in cancer growth and metastasis” is still not fully understood (Shvartsur and Bonavida 2015). In our observational study, we cannot provide evidence on further molecular mechanisms, particularly in tumors displaying temporal changes in TROP-2 expression. Consequently, further mechanistic investigations are warranted to elucidate the determinants of TROP-2 expression. A deeper understanding of potential dynamics of TROP-2 expression and their underlying mechanisms may prove clinically relevant, e.g., in the context of a potential future use of a TROP-2-directed ADC in the adjuvant setting or in rechallenge scenarios involving alternative TROP-2-targeting agents at later disease stages.

### Limitations

Our study has several limitations. First, the analysis was conducted in a monocentric setting. Second, the assessment of TROP-2 expression by IHC is inherently semi-quantitative and may be influenced by tissue-specific factors, such as fixation, decalcification, and small biopsy size, and may leave room for intra- and interobserver variability. As such, minor changes in the H-score may be arbitrary, and care should be taken not to infer a level of precision that exceeds the methodological limitations of the analysis. Intra- and interobserver variability may be overcome by new approaches, such as the quantitative continuous scoring (QCS) mentioned above. However, these approaches may not correct tissue-inherent bias. Moreover, the value of a scoring method that can be quickly and cost-effectively implemented anywhere should not be underestimated. Third, time points were defined by clinical availability rather than a prespecified study schedule; therefore, TP1–TP4 do not correspond to identical treatment lines in all patients. Fourth, receptor-conversion and TROP-2-change analyses may be affected by spatial sampling bias, as many paired comparisons involved different anatomical sites or different metastatic organs. Fifth, the SG subgroup was small and biologically selected by the need for SG treatment and the availability of paired tissue. Therefore, decreases in TROP-2 after SG may represent treatment selection pressure, adverse baseline biology, sampling differences, or a combination of these factors. Finally, selected clinicopathological parameters were not available for all metastatic biopsies. In particular, Ki-67 was not routinely assessed in metastatic specimens in our practice, as it rarely influences therapeutic decision-making in this setting. Moreover, in a subset of cases, pathological assessment was difficult or even impossible due to limited tissue quantity and quality, with insufficient material for additional stainings. As a result, Ki-67 and, in some cases, receptor status parameters (HR and/or HER2) were unavailable for some metastatic samples. The small SG sample size also precluded robust adjustment for previous therapy lines, receptor subtype, or biopsy organ. Nevertheless, the present study provides clinically relevant real-world insights into longitudinal TROP-2 expression dynamics in mBC.

## Conclusion

TROP-2 expression was generally high and relatively stable across different clinically indicated time points throughout the treatment journey of individual BC patients, which is consistent with the evidence base supporting current regulatory approvals of TROP-2-directed ADCs, irrespective of routine TROP-2 testing. Importantly, these time points reflected clinically driven diagnostic and reassessment procedures rather than uniform treatment-line intervals. We did not observe a relevant overall difference in TROP-2 expression between local/primary and metastatic samples. However, some patients in our series showed a decrease or even loss of TROP-2 expression after receiving several lines of therapy. In the small SG-paired subgroup, pre-SG samples were collected prior to the first administration of SG, and post-SG samples after SG exposure; all representative last pre-SG and first post-SG specimens originated from metastatic lesions. Although a decrease in TROP-2 expression was observed in some patients after SG, the paired SG analysis did not reach statistical significance and may reflect treatment-related selection pressure, aggressive disease biology, heterogeneous previous therapy exposure, spatial sampling effects, or a combination of these factors rather than a direct causal mechanism. Accordingly, these findings should be interpreted as descriptive and hypothesis-generating. In view of emerging, broader, and potentially sequential TROP-2-targeting treatment options, analysis of TROP-2 expression in larger, prospectively sampled cohorts would be useful to better understand underlying mechanisms and to fully exploit the clinical potential of these agents.

## Data Availability

The datasets generated and analyzed during the current study are available from the corresponding author on reasonable request.
